# The effect of temperature on the rate of oxygen evolution reaction during ferrate(VI) synthesis by anodic dissolution of iron in highly alkaline media

**DOI:** 10.1016/j.heliyon.2024.e35414

**Published:** 2024-07-30

**Authors:** Javier Quino-Favero, Abel Gutarra Espinoza, Erich Saettone, Juan Carlos Yacono Llanos, Fabricio Paredes Larroca

**Affiliations:** Universidad de Lima, Facultad de Ingeniería Industrial, Av. Javier Prado Este s/n, Monterrico, Lima, Peru

**Keywords:** Ferrate(VI), Oxygen evolution reaction, OER, Space-time yield, Current efficiency

## Abstract

This study investigates the effect of temperature on the rate of the oxygen evolution reaction (OER) during the electrochemical production of ferrate(VI) through anodic iron dissolution. We employed a membrane-divided electrochemical cell with a galvanostatically operated three-electrode setup. During the experiments, we recorded the anode potential at various temperatures and monitored temperature variations over time. Simultaneously, we measured the rates of ferrate(VI) formation and the oxygen evolution reaction. The latter, considered a parasitic reaction, competes with ferrate synthesis. By quantifying the extent to which the OER consumed the applied charge, we discovered that the OER rate decreases with temperature. Specifically, at 25 °C and 168 Am^−2^, the OER consumes more than double the charge of the produced ferrate, at higher temperatures the rate sensibly decays and with it the consumed charge by the OER. The specific energy required for ferrate(VI) production decreases as temperatures increase, aligning well with current efficiency and space-time yield values within the same temperature range.

## 1 Introduction

Tetraoxy high-valent iron anions, commonly called ferrates, are species where the iron valence state is higher than the +2 and + 3 oxidation states of which the most stable is +6 or FeO_4_^−2^ [1]. Ferrate(VI) exhibits a high oxidant potential due to the redox potential that ranges from +2.20 V to +0.72 V in acidic and alkaline solutions [2]. As ferrates oxidize substances, they reduce to Fe(III) ions that act as a coagulant; this behavior has a high potential in water treatment because ferrates act as multifunctional agents that can be used to disinfect, partially degrade organic pollutants, oxidize inorganic ions, and remove colloidal particulates [3] with a single dose and mixing unit process [4]. As ferrate(VI) reduces it forms harmless Fe(III) ions and has been dubbed as an ecologically friendly oxidizer [5].

Ferrate(VI) can be synthesized using three methods: wet oxidation, dry oxidation and electrosynthesis [6]. In the wet oxidation method, Fe (III) ions are oxidized to Fe(VI) ions using hypochlorite in a concentrated alkali solution [[7], [8], [9], [10]]; In the dry oxidation method, iron filings are reacted with group I alkali hydroxides and peroxides in a high temperature environment to synthesize ferrate(VI) [[11], [12], [13]]. In ferrate (VI) electrosynthesis, an iron-based anode material in a strong alkali solution undergoes oxidation in the superpassivation potential region that dissolves to form ferrate(VI) [6,[14], [15], [16], [17]].

Ferrate (VI) electrosynthesis is usually carried out in a two-component electrolysis cell where the anodic and cathodic chambers are separated with an ion exchange membrane [14,18,19], ferrate(VI) yield is strongly related to anode material, separator, type and concentration of alkaline electrolyte, anodic current density, temperature, dissolved oxygen and electrolysis time [[20], [21], [22], [23], [24], [25]].

During the electrochemical synthesis of ferrate(VI) using a dissolving iron anode in a highly alkaline electrolyte, a current is used to oxidize iron to ferrate (VI) in a process that can be summarized as a three-step process: formation of intermediate species, formation of ferrate (VI) and passivation of the iron electrode, where passivation describes the formation of a layer of oxides and hydroxides that prevent further generation of ferrate(VI) [26]; to continue the production of ferrate(VI), the passivation layer must be continuously dissolved through the interaction of the hydroxyl ions of the electrolyte.

The applied current density and time of application affect the efficiency of ferrate formation and are related with the applied potential, which in turn allows reactions to occur at the electrode surface. As ferrate(VI) formation occurs in the transpassive region it competes with the oxygen evolution reaction, and this consumes a portion of the current that could be used for ferrate production, resulting in a lower yield of ferrate(VI) [27]. Ferrate synthesis requires a high concentration alkaline medium like NaOH 16 mol/L [28], 14–16 mol/L [29] or 20 mol/L [26]. The surface layer of the anode is influenced by both the electrolyte concentration and the composition; using a more concentrated OH^−^ solution, both the surface layer disintegration and the ferrate(VI) stability are increased [27].

Temperature serves as a crucial operational factor that is related to two opposing impacts, an increase in temperature generates depassivation of the electrode surface, improving the chemical interaction of the oxo-hydroxide layer with the hydroxyl anions of the electrolyte, increasing ferrate(VI) production, and promoting ferrate(VI) decomposition kinetics [27].

The specific energy consumption, kWh per kg of generated ferrate, is variable as it depends on several other variables like electrolyte type and concentration, electrode material, spacing between electrodes, temperature, and current density. Specific energy consumption values for ferrate(VI) have been reported from 1,2 kWh/kg [30] to 6 kWh/kg [31] which is within the range of other oxidants like ozone with 2,5–10,4 kWh/kg [32,33] and chlorine 4,8–5,5 kWh/kg [34].

Compared to wet and dry oxidation methods, the electrochemical methods are simple in process, operation and results in a high purity product; however, these methods are not exempted from issues that need to be addressed such as electrode passivation, competitive oxygen evolution, demanding electrolyte and alkaline requirements, and significant energy usage [33].

In this study, we employed a membrane-divided electrochemical cell with a galvanostatically operated three-electrode setup. This setup allowed us to continuously monitor ferrate synthesis and oxygen mass flow while simultaneously recording the anode potential at various temperatures and during temperature variations over time. We report standard variables such as current yield and specific energy consumption for comparison with other published studies. To the best of our knowledge, this study marks the first direct measurement of oxygen evolution during electrochemical ferrate production. Given that the oxygen evolution reaction (OER) competes with ferrate synthesis and influences yield, we investigated how the temperature affects the oxygen evolution rate and its potential impact on ferrate production efficiency.

## 2 Material and methods

### 2.1 Description of the Fe(VI) reactor

**Fig. 1:**
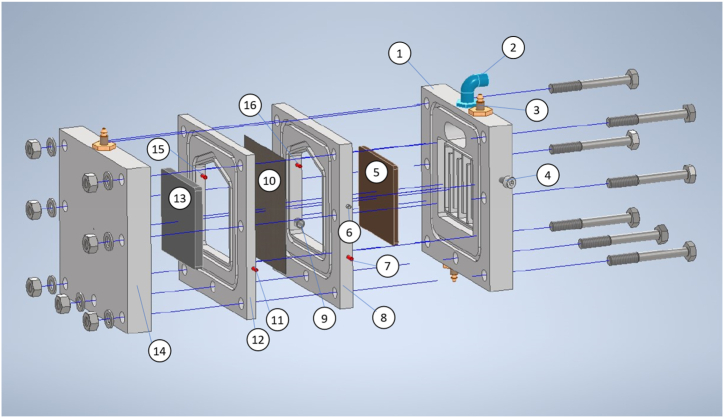
Fe(VI) reactor and parts. (1) Anode chamber cover, (2) Water outlet, (3) Threaded male connector for O_2_ collection, (4) Anode electrical contact, (5) Iron anode, (6) Thermocouple sheath, (7) anolyte outlet port, (8) anodic chamber (9) Reference electrode port, (10) Ion exchange membrane, (11) catholyte outlet, (12) cathodic chamber, (13) graphite cathode, (14) Cathode chamber cover, (15) catholyte inlet (16) anolyte inlet.

The Fe(VI) reactor (Fig. 1) is an electrolytic cell where the anode is a 4.8 cm × 4.8 cm x 0.4 cm (length x height x width) iron plate (99.65 % iron, 0.0470 % carbon and 0.0545 % silicon content, composition was determined by mass spectrometry using a Spectro 1 Spectrolab M8 arc spark optical emission spectrometer), the cathode is a graphite plate of the same dimensions, both separated by 5 cm. An ion exchange membrane CTIEM-1 (Perfluorosulfonic Acid Cation Exchange Membrane Zibo Cantian, China) which is used as an ion exchange membrane for the chlor-alkali industry, 2.3 Ω cm^2^, is placed between the electrodes. The membrane was selected due to its resistance to strong alkali solutions and working temperatures around 70–75 °C according to manufacturer data, initial voltages remained constant before each test, indicating minimal changes in the conductivity of the cell and therefore of membrane integrity. The electrodes and the membrane are fixed and encapsulated in polymethyl methacrylate (PMMA) supports and separators, maintaining an A/V ratio of 1.40 m^-1^ in the anodic chamber. The electrolyte is a 20 mol dm^−3^ solution of NaOH, with a volume of 16.5 × 10^−3^ dm^3^ in the anodic chamber and 13.5 × 10^−3^ dm^3^ in the cathodic chamber. The top of the anodic chamber is sealed and has an opening for collecting the evolved oxygen. Additionally, the reactor has connectors with hoses to charge NaOH and discharging Fe(VI), a sheath to place a PT100 thermocouple that measures the anolyte temperature, and a port for placing the Hg/HgO reference electrode (Koslow Scientific 5088 Series, USA) at a distance of 1 mm from the anode. The reactor also has a chamber where water circulates in contact with the back face of the anode, allowing temperature control.

### 2.2 Experimental setup

**Fig. 2:**
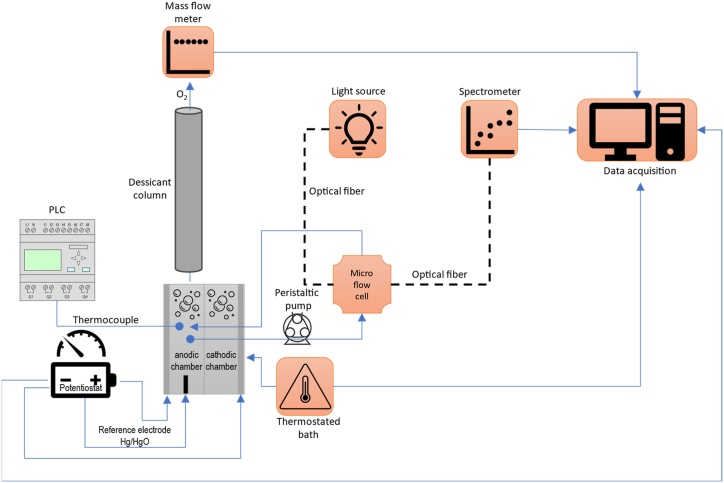
Experimental setup.

The system comprises an electrochemical cell and a set of instruments that allow data recording (Fig. 2). The ferrate(VI) reactor is powered by a potentiostat (Autolab PGSTAT302N, The Netherlands), which provides constant current and simultaneously measures the voltage of the working electrode (anode) relative to a Koslow Scientific 5 mm PTFE/PP mercury oxide electrode (New Jersey, USA). Water that circulates in contact with the anode is temperature controlled using a thermostatic recirculatory bath with ±0,1 precision (Daihan Scientific, Korea). The anolyte temperature is measured using a PT100 thermocouple connected to a Novus TxRail-USB transmitter, which communicates with a Siemens Simatic S7-1200 PLC.

The rate study of ferrate(VI) production is conducted using a peristaltic pump (Lead Fluid BT50S, China) that continuously extracts the anolyte and directs its flow to a stainless steel microflow cell (Custom Sensors and Technology, Fenton, USA) with 1/8″ Micro Optical Interface Couplers (mOICs) with fused silica intern optics to continuously monitor the solution's absorbance (and consequently, the concentration) before returning to anodic chamber were it also provide mixing. The flow cell is connected to two 600 μm optical fibers, one connected to the DH Mini Deuterium Halogen Light Source and the other to a Flame XR spectrophotometer, both using OceanView software (all from Ocean Optics, Orlando, USA). The O_2_ generated in the anodic chamber passes through a column with anhydrous CaCl_2_ to remove water before entering an Alicat M-50SCCM-D-485 mass flow meter (Alicat Scientific, Tucson, USA). The data measured by each of the sensors is recorded and saved on a computer with FlowVision Software 2.0.

### 2.3 Calculations

The concentration (CFe(VI)) of produced Fe(VI) is calculated using the Beer-Lambert law based on the recorded absorbance (A) and utilizing a molar extinction coefficient of 1050 L mol^−1^ cm^−1^ adjusted to the optical interfaces' separation (d). This is expressed by equation (1).

(1) $$C_{Fe\left(VI\right)}=\frac{A}{\varepsilon .d}$$

The current yield (F_Eff_) is calculated based on the amount of electric charge required for the formation of Fe(VI) (QFe(VI)) or O_2_ (QO_2_) in relation to the electric charge provided to the electrolytic reactor (Q_rea_). This is expressed by equations (2), (3).

(2) $${FEff}_{Fe\left(VI\right)}=\frac{Q_{Fe\left(VI\right)}}{Q_{rea}}\times 100$$

(3) $${FEff}_{O_{2}}=\frac{Q_{O_{2}}}{Q_{rea}}\times 100$$

If the concept of specific energy (SEn) is applied to the production of ferrate(VI) and O_2_, the amount of electric energy (V.I.t) required per unit mass (m) produced is obtained. This is expressed by equations (4), (5).

(4) $${SEn}_{Fe\left(VI\right)}=\frac{{\left(V.I.t\right)}_{Fe\left(VI\right)}}{m_{Fe\left(VI\right)}}$$

(5) $${SEn}_{O_{2}}=\frac{{\left(V.I.t\right)}_{O_{2}}}{m_{O_{2}}}$$

Space-time yield (STY) is the amount of ferrate produced per unit of time and reactor volume. It is proportional to current density, current efficiency and the working surface area of electrode per unit of volume [31]. STY is expressed by equation (6).

(6) $$S\;TY=\frac{m_{Fe\left(VI\right)}}{V_{anolite}.t}$$

## 3 Results

### 3.1 Effect of temperature in the rate of ferrate(VI) formation

The rate of ferrate formation increases with increasing temperatures as have been previously reported [23,35]. Higher temperatures produce a depassivation of the electrode surface, caused by an enhancement of the reaction of the alkaline electrolyte with the oxyhydroxide layer, which exposes fresh layers of the electrode that continue to experience an anodic dissolution, improving the production yield of ferrate(VI) (Mácová et al., 2009)Simultaneously and oppositely, the temperature increase accelerates the ferrate decomposition kinetics (Machala et al., 2008), which is marked when the ferrate concentration is high because the decomposition of ferrate associated with higher temperatures follows first order kinetics when pH is above 10 (Cataldo-Hernández et al., 2018; Lee & Gai, 1993).

**Fig. 3:**
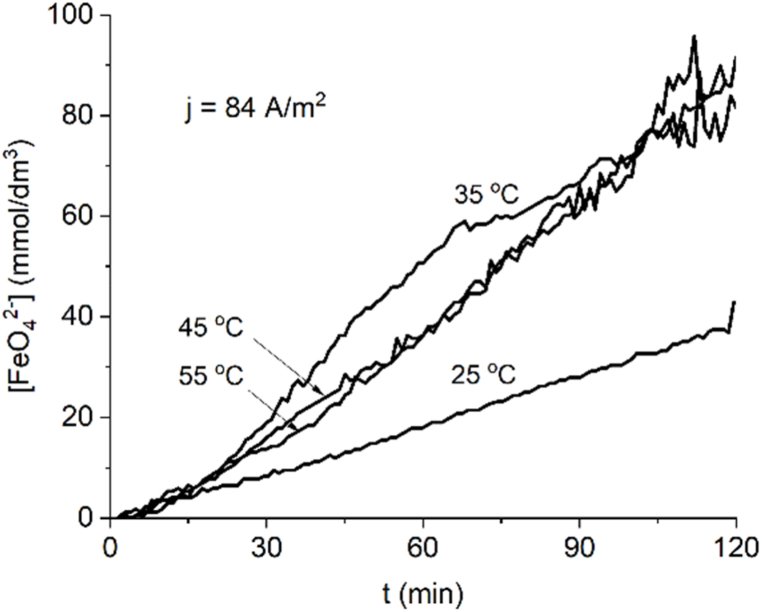
Ferrate(VI) formation at a current density of 84 Am^−2^ at several temperatures.

**Fig. 4:**
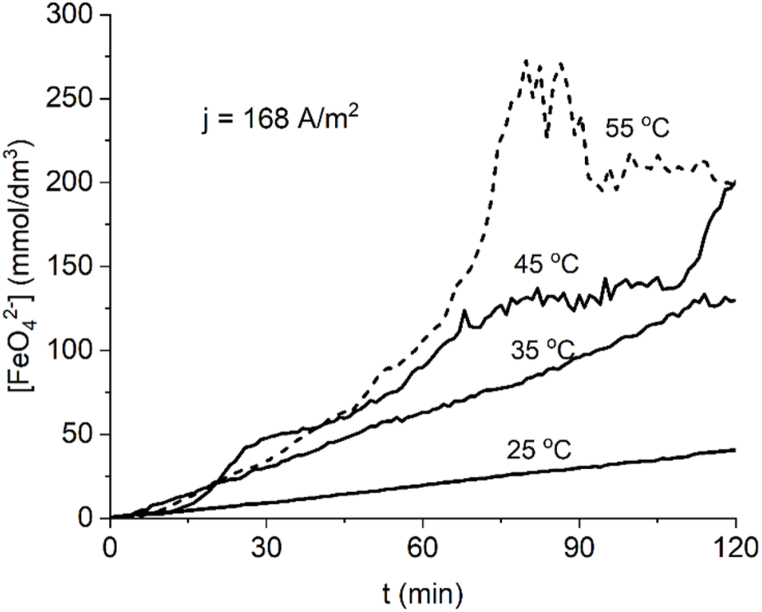
Ferrate(VI) formation at a current density of 168 Am^−2^ at several temperatures.

At the lowest current density of 84 A/m^2^ (Fig. 3) the rate of ferrate formation is lower than at 168 A/m^2^ (Fig. 4) because less charge is transferred. Fig. 3 does not show an increase in rates at increasing temperatures, as expected, because ferrate thermal decomposition at 45 and 55 °C appears to be higher enough to negatively impact rates at the higher temperatures. The effect is not seen at a higher current density of 168 A/m2 (Fig. 4) where the ferrate production rates are high enough to offset, at least temporarily, the thermal decomposition of the ferrate.

At the higher limit of the carried out essays (55 °C, Fig. 4) a rapid increase in ferrate (VI) concentration can be seen up to 75 min, and after this lapse the measurements become highly unstable and are followed by a loss in absorbance which is interpreted as the thermal decomposition of ferrate(VI) in the electrolyte solution.

### 3.2 Dependence of ferrate current yield on the temperature with time

**Fig. 5:**
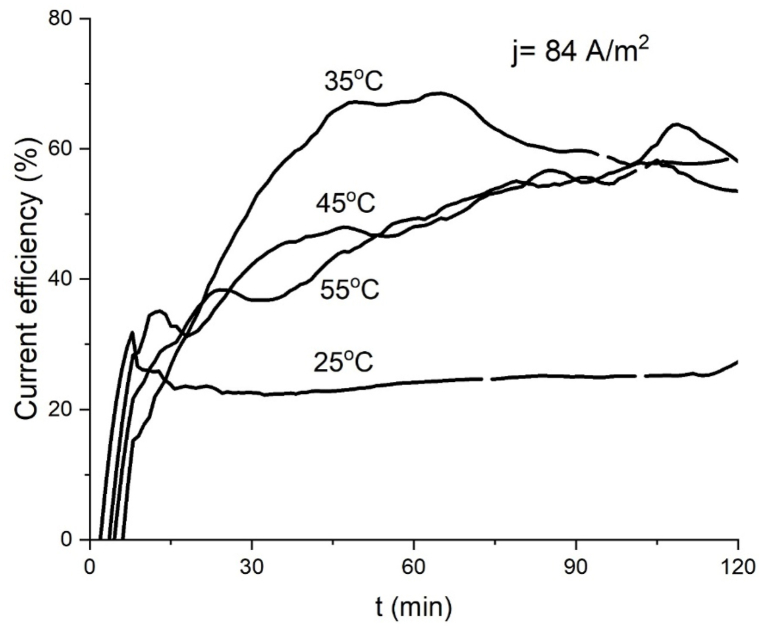
Current efficiency for ferrate (VI) at J = 84 Am^−2^ at several temperatures.

**Fig. 6:**
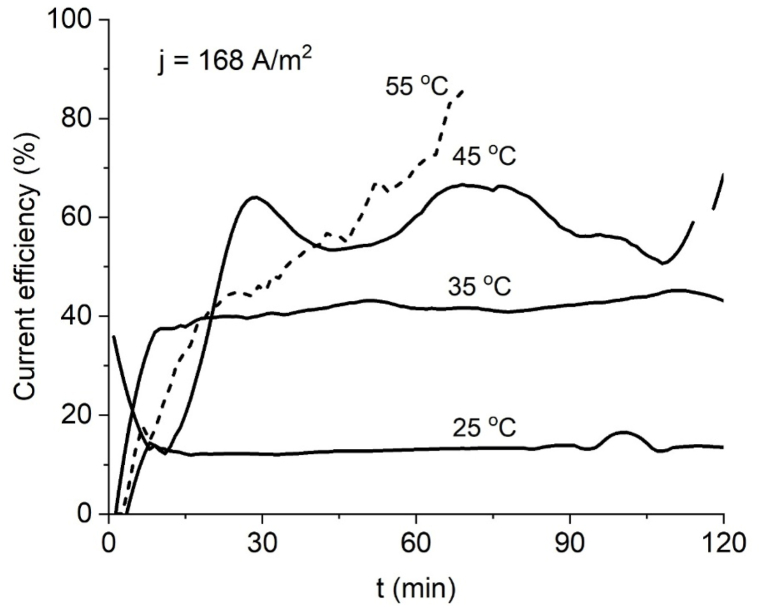
Current efficiency for ferrate (VI) at J = 168 Am^−2^ at several temperatures.

Being an electrochemical driven reaction, ferrate(VI) current yield depicts the quotient between product formed and product potentially formed, because of this the curves of yield versus time for several temperatures at constant current density reflect the progress of ferrate production with time because theoretical yield is constant. Current efficiency curves are mostly constant when the rate of ferrate production tends to be linear, like the curves produced at 25 °C at 84 Am^−2^ (Fig. 3) and 25 °C, 35 °C at 168 Am^−2^ (Fig. 4). The final current efficiency at 84 A m^−2^ for 35, 45 and 55 °C is 53 %, 58 % and 59 % after 120 min (Fig. 5); the final current efficiency at 168 Am^−2^ is 69 % for 45 °C (Fig. 6). At 55 °C the current efficiency calculation is unstable due to the variable absorbance measurement. At low currents and temperatures, the concentration of the intermediate products for ferrate(VI) synthesis in the reaction layer is low and production and yield are low (Bouzek & Roušar, 1993). Table 1 shows a comparison with other work using the same type and concentration of electrolyte, higher realized concentrations by this work are attributed to cell design.

**Table 1:** Ferrate concentrations, efficiencies, and specific energy consumption for several works using NaOH 20 mol dm^−3^ as electrolyte (ns = not stated).

Current density Am^−2^	NaOH mol/L	Anode iron content %	FeO_4_^−2^ mmol/dm^3^	Efficiency (%)	EC kWh kg^−1^	Time (min)	Reference
14.7	20	99.195	1.74	89.5	2.616	90	Barisci 2014[26]
36	20	ns	2.16	43	6.3	25	Alsheyab 2010 [31]
168	20	99.65	200	68	9	120	This study
1000	20	ns	57.5	ns	2.01	ns	Castañeda 2020 [36]
ns	20	99.195	2.26	>70	<2.9	90	Barisci 2018[37]

### 3.3 Space-time yield (STY), current efficiency and specific energy

**Fig. 7:**
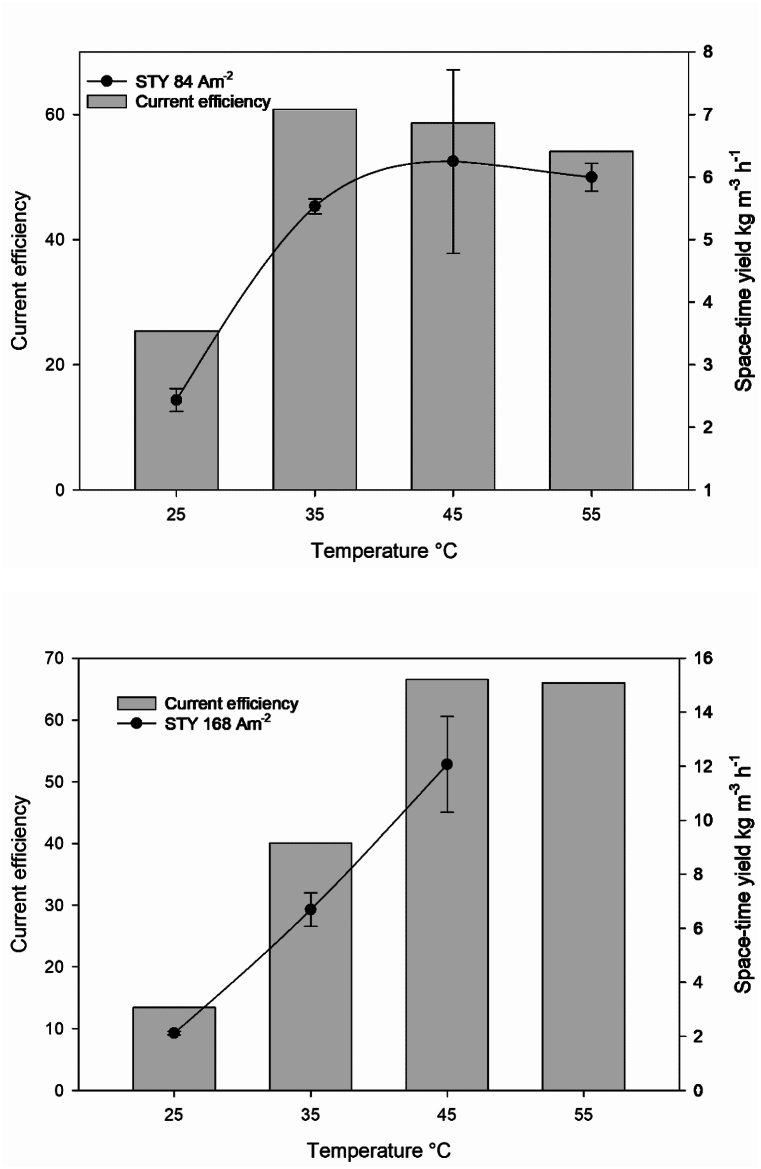
Space-time yields and current efficiencies for ferrate(VI) production at J = 84 Am^−2^ (up) and J = 168 Am^−2^ (down).

**Fig. 8:**
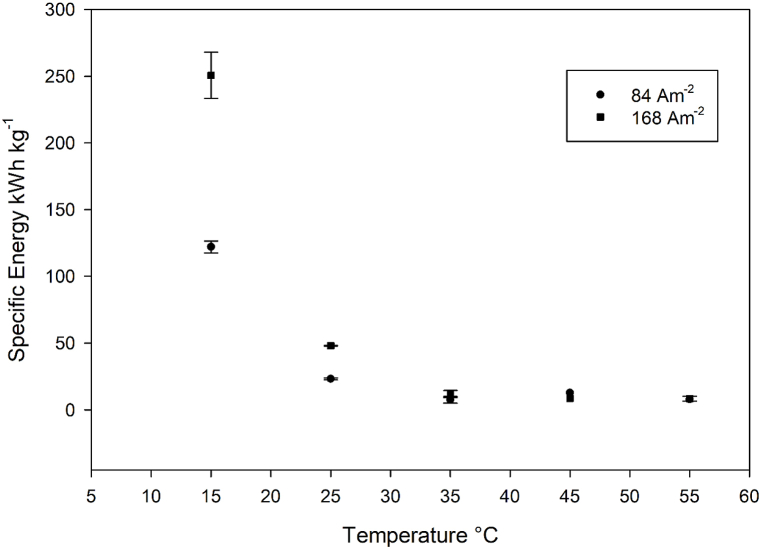
Specific energy consumption calculated at the end of synthesis (120 min).

We calculated the space-time yield (STY) based on ferrate production progress curves over time, at four different temperatures (Fig. 7). At 45 °C, the maximum STY values were 6 kg m^−3^ h^−1^ (at a current density of 84 Am^−2^) and 12 kg m^−3^ h^−1^ (at a current density of 168 Am^−2^). However, the ferrate(VI) progress curves at 55 °C exhibited erratic behavior, as seen in Fig. 4, making it challenging to calculate STY accurately for this temperature. Consequently, the STY at 55 °C is not included in Fig. 8. A previous report [31] have highlighted the impact of the anode-to-volume ratio (A/V) on ferrate generation efficiency during the reaction. Specifically, a higher A/V ratio leads to more efficient ferrate production. This relationship arises because, for the same volume of anolyte, the ferrate production is directly proportional to the anode's surface area.

Specific energy consumption in kWh kg^−1^ decreases with increasing temperatures (Fig. 8), and the difference in the two tested current densities decreases at higher temperatures. Specific energy consumption is well in line with current efficiency and space-time yield for the same temperature range.

### 3.4 Effect of temperature in the rate of oxygen formation

The electrochemical synthesis of ferrate(VI) via anodic oxidation of an iron electrode in strongly alkaline media represents the most common method for the generation of ferrate(VI) [1,16,21,[26], [27], [28], [29],[38], [39], [40]]. However, researchers have pointed out a significant limitation: the parasitic oxygen evolution reaction (OER) [18,27,33,39,[41], [42], [43]]. This reaction occurs when the high electrical potential used to generate ferrate(VI) exceeds the stability of water (approximately 0.8 V vs. SHE at pH = 7), leading to a decrease in current efficiency [41]. Furthermore, studies by Mácová et al. have highlighted that fast reaction kinetics in the transpassive potential region contribute to OER enhancement [27]. To mitigate the impact of anode material and OER, researchers have proposed several strategies. These include using electrodes with high oxygen overpotential [18,41,44], such as boron-doped diamond (BDD), and the use of molten hydroxides as an electrolytic environment [45,46].

**Fig. 9:**
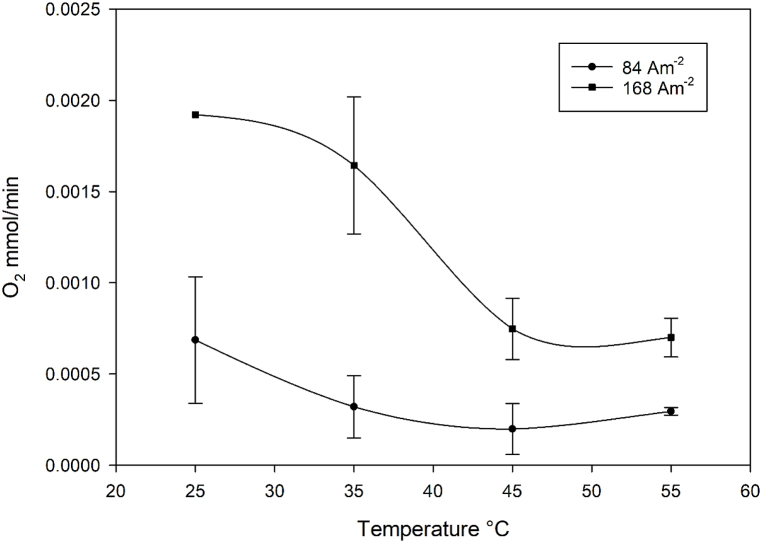
Rates of oxygen evolution versus temperatures during ferrate(VI).

To assess the impact of the oxygen evolution reaction (OER) on ferrate synthesis, we measured the oxygen evolution rates during the time of electrolysis. Interestingly, we observed that the OER rate decreased as the temperature increased (as shown in Fig. 9), this trend is observed up to 55 °C at 168 A/m^2^ and up to 45 °C at 84 A/m^2^. At 84 A/m^2^ and 55 °C the mean of oxygen evolution rate have a slight increase, but their values and deviations are within the standard deviations of the rates at 45 °C and it cannot be said whether the effect is real or just part of the error in the measurements.

The decrease in the OER is more pronounced at the high current density of 168 A/m^−2^ that we tested. To better understand the contribution of each reaction, we calculated the faradaic charge at 100 min and 38 min (times chosen to work with the linear part of ferrate production at 45 and 55 °C), for both oxygen and ferrate formation, determining their respective shares relative to the total faradaic charge applied.

**Fig. 10:**
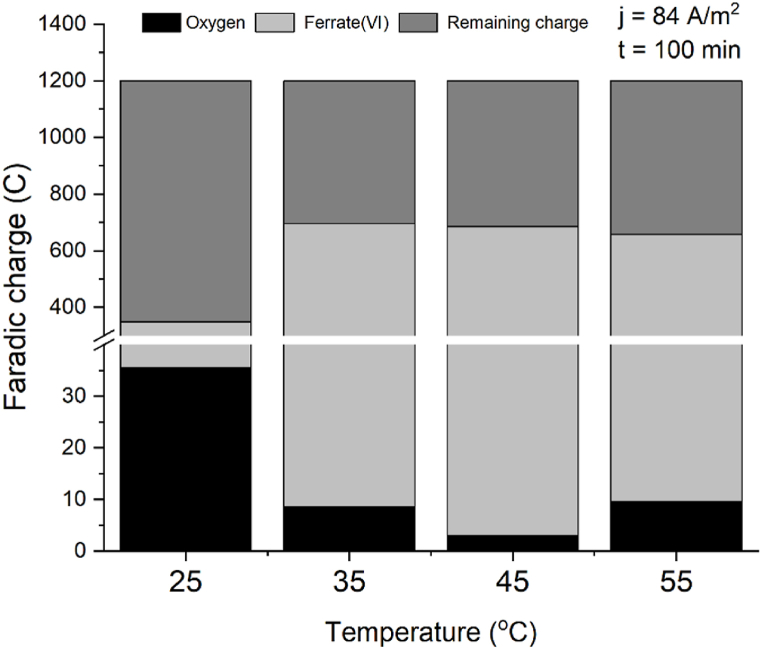
Shares of oxygen and ferrate(VI) charge consumed relative to the total faradaic charge applied after 100 min of electrolysis at current density of 84 Am^−2^.

**Fig. 11:**
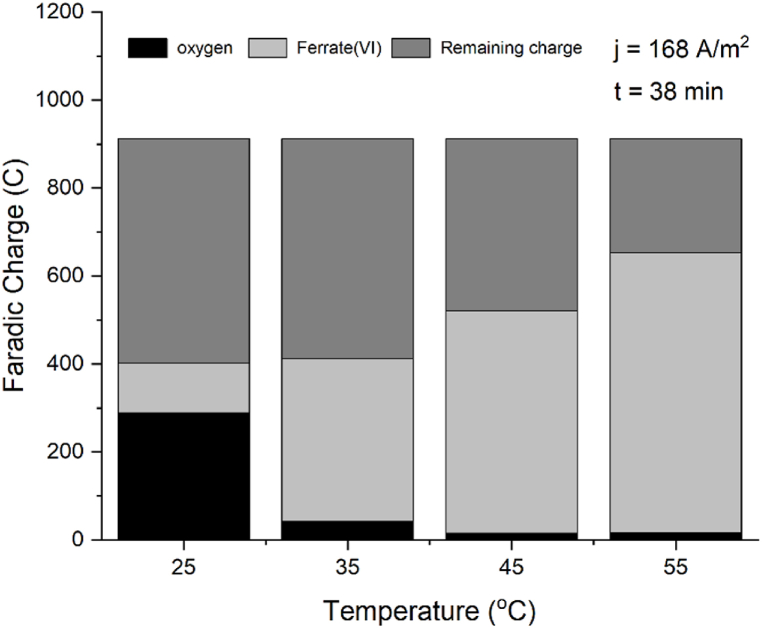
Shares of oxygen and ferrate(VI) charge consumed relative to the total faradaic charge applied after 38 min of electrolysis at current density of 168 Am^−2^.

At 168 A/m^2^ and increasing temperatures the charge consumed for oxygen decreases while for ferrate increases (Table 2 and Fig. 11). At 84 A/m^2^ the same trend holds until 45 °C. At 55 °C the thermal decomposition is higher which traduces in less produced ferrate, and simultaneously the slight increase in the oxygen evolution rate (as seen in Fig. 9) lead to an increased charge for oxygen and a decreased charge for ferrate, which traduces in a smaller ratio FeO_4_^−2^:O_2_ (as seen in Fig. 10 and Table 2).

**Table 2:** Ratios of shares of charge for oxygen and ferrate(VI) at two current densities.

T °C	J = 84 Am^−2^ Q_total_ = 600C			J = 168 Am^−2^ Q_total_ = 1200C		
	O_2_(C)	FeO_4_^2−^(C)	ratio FeO_4_^2−^:O_2_	O_2_(C)	FeO_4_^2−^(C)	ratio FeO_4_^2−^:O_2_
25	36	313	9	288	114	0.4
35	9	688	76	42	370	9
45	3	682	227	15	506	34
55	10	648	65	16	637	40

At 25 °C and 84 Am^−2^ (Fig. 10) the charge consumed by ferrate synthesis is 9 times the required for the OER, this ratio increases with the increment of the temperature. It is notable that in the case of 25 °C and 168 A/m^2^ (Fig. 11) the electric charge used for oxygen is more than double than what is used for the synthesis of ferrate, under these operating conditions the system is totally inefficient. At higher temperatures the charge consumed by the OER is a small fraction of the consumed by ferrate(VI) synthesis. Results are summarized in Table 2.

### 3.5 Relationships between anode potential and oxygen and ferrate rates at continuously varying temperature

Conducting experiments at constant temperature is beneficial for studying the kinetics of ferrate and oxygen production within a specified time-frame. However, we were also curious to know whether the relationships observed in previous experiments would hold true when applying an increase and subsequent decrease in temperature.

**Fig. 12:**
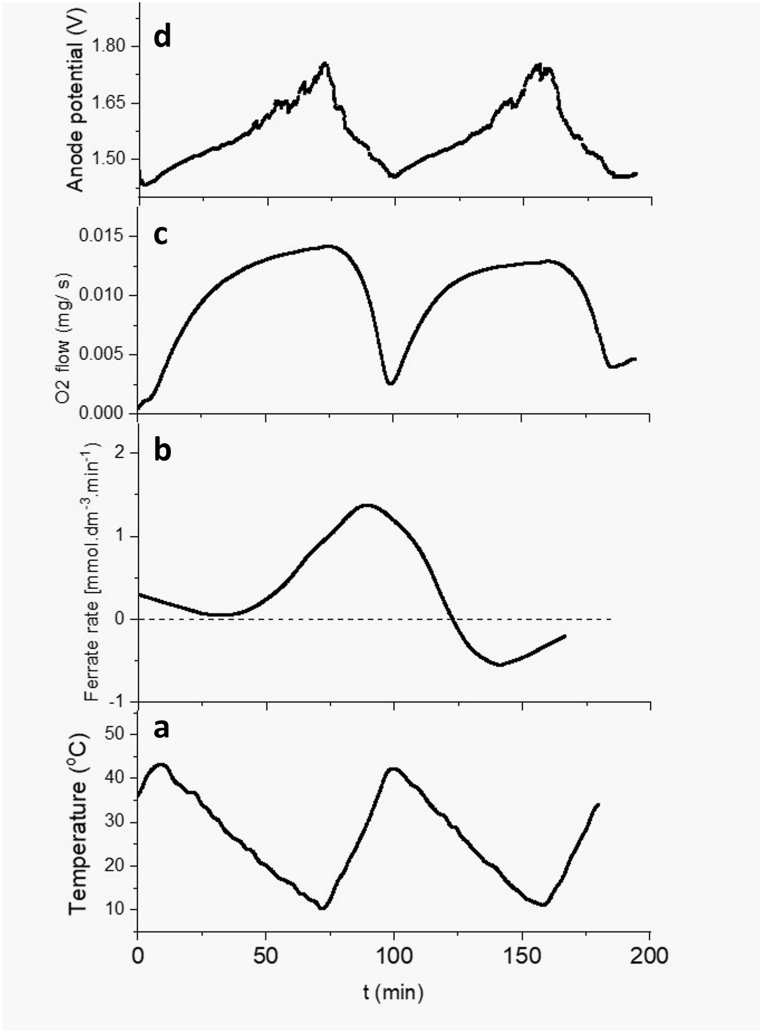
Temporal evolution of: (a) temperature, (b) ferrate(VI) production rate, (c) oxygen evolution flow, and (d) anode potential.

With the aim of this study in mind, a heating-cooling ramp was implemented in the experimental setup, spanning a period of approximately 90 min, and ranging between 10 °C and 45 °C, as depicted in Fig. 12(a). Throughout this temperature variation, concurrent measurements were taken for the ferrate(VI) production rate, gaseous oxygen flow, and the anode potential relative to the reference electrode. Fig. 12(b) illustrates the ferrate(VI) production rate, revealing the rate of ferrate(VI) generation. Notably, there appears to be a delay in comparison to the temperature curve. This delay can be attributed to the fact that ferrate is not primarily produced at the surface of the electrode but rather within the bulk of the solution. The complex reactions involved in the electrochemical ferrate(VI) synthesis have limiting steps unrelated to electron transfer. For instance, the dissolution of the oxyhydroxide layer in alkaline media (equation (7)) or the disproportionation reaction play crucial roles in ferrate production (equation (8)) [27]:

(7) $$FeOOH+OH^{-}\to FeO_{2}^{-}+H_{2}O$$

(8) $$e^{-}+3FeO_{3}^{2-}+H_{2}O\;\to 2FeO_{2}+FeO_{4}^{2-}+2OH^{-}$$

Around 120 min later, the rate shifts to negative values, potentially indicative of ferrate(VI) degradation. Fig. 12(c) shows the inverse relationship between the flow of oxygen generation and temperature changes, consistent with previous observations. Similarly, the anode potential curve (Fig. 12(d)) exhibits an inverse correlation with temperature, aligning with the proposed dissolution of the anode passivating layer as temperature increases, as suggested by prior research.

## 4 Conclusions

The oxygen evolution reaction (OER) is often considered a parasitic reaction that competes with ferrate(VI) synthesis during the anodic dissolution of iron in highly alkaline media. Our findings reveal that the OER rate decreases with increasing temperature at both tested current densities, with the exception at 55 °C and J = 84 Am^−2^ where thermal decomposition diminishes the charge consumed for ferrate. Specifically, at J = 168 Am^−2^ and 25 °C, the OER reaction consumes more than 2.5 times the charge consumed by ferrate. However, this ratio decreases significantly at higher temperatures. Therefore, it is recommended to work at elevated temperatures until a limit of 55 °C, beyond which the decomposition of the ferrate becomes pronounced. The maximum space-time yield achieved was 12 kg m^−^³ h⁻^1^ at 186 A m⁻^2^, with a current efficiency of 66.5 % and a specific energy of 8.56 kWh kg⁻^1^. In particular, the cell design, featuring a high Surface:Volume ratio of 1.4 where the produced ferrate can be dissolved in a low volume of electrolyte, allows the production of high concentrations of ferrate(VI), reaching up to 200 mmol dm^− 3^ - an order of magnitude higher than reported in similar studies.

## Data availability statement

The data presented in this study are openly available in FigShare at https://figshare.com/10.6084/m9.figshare.25557594.

## CRediT authorship contribution statement

**Javier Quino-Favero:** Writing – review & editing, Writing – original draft, Methodology, Investigation, Formal analysis, Conceptualization. **Abel Gutarra Espinoza:** Writing – review & editing, Writing – original draft, Methodology, Investigation, Formal analysis. **Erich Saettone:** Writing – review & editing, Writing – original draft, Methodology, Investigation, Formal analysis. **Juan Carlos Yacono Llanos:** Writing – review & editing, Writing – original draft, Investigation. **Fabricio Paredes Larroca:** Software, Methodology, Investigation.

## Declaration of competing interest

The authors declare that they have no known competing financial interests or personal relationships that could have appeared to influence the work reported in this paper.
